# Using Ecological Momentary Assessment to Assess Family Functioning in Spanish-Speaking Parent and Adolescent Dyads: Daily Questionnaire Study

**DOI:** 10.2196/60073

**Published:** 2025-06-11

**Authors:** Alejandra Fernandez, Savannah Bernal, Lana Kim, Subodh Potla

**Affiliations:** 1 Department of Social and Behavioral Sciences, Peter O'Donnell Jr. School of Public Health, The University of Texas Southwestern Medical Center, 5323 Harry Hines Blvd, Dallas, TX 75390, United States, 1 2146452533; 2 Department of Health Promotion and Behavior Sciences, UTHealth Houston School of Public Health, Dallas, TX, United States; 3 Department of Integrative Biology, Rice University, Houston, TX, United States; 4 Deparment of Epidemiology, UTHealth Houston School of Public Health, Dallas, TX, United States

**Keywords:** adolescence, ecological momentary assessment, family functioning, family function, feasibility, acceptability, adolescent, family-based, family-based interventions, EMA, community-based, smartphone

## Abstract

**Background:**

Family functioning is associated with several adolescent health outcomes, and many family-based interventions (FBIs) exist to improve family functioning. However, most FBIs assess family functioning retrospectively at baseline and post intervention, thereby overlooking the daily fluctuations in family functioning throughout the intervention. Ecological momentary assessment (EMA) is a method involving a high frequency of assessments and has been underused to assess family functioning across parent and adolescent dyads. Further, limited research exists on the use of EMA in bilingual populations.

**Objective:**

The purpose of this study was to assess an EMA protocol’s feasibility and acceptability and to analyze within-person and between-person variance in family functioning reports in a sample of primarily Spanish-speaking parent and adolescent dyads.

**Methods:**

Participants completed a baseline assessment (including demographics and family functioning assessment), a 7-day protocol with a once-daily family assessment questionnaire using an EMA app, and an acceptability questionnaire at the conclusion of the study.

**Results:**

We recruited 7 mothers (mean age 37.29, SD 3.82 years) and 8 adolescents (n=7, 88% females; mean age 11.86, SD 1.07 years) who identified themselves as Hispanic/Latinx. The participants showed overall satisfaction with the EMA protocol. The daily assessments were completed relatively quickly (mean 3 minutes and 16 seconds, SD 11 minutes and 5 seconds) after the prompt notification was received, and the response rate across the daily assessments was 90% (87/97). The reported family functioning was relatively high across both adolescents (mean 4.57) and parents (mean 4.59). The variance across adolescents (SD 0.459) was larger than that within their individual reports of family functioning (SD 0.122). Alternatively, the variance across parents was smaller (SD 0.132) than that reported among parents’ individual reports of family functioning (SD 0.286). Our findings highlight the heterogeneity between adolescent and parent responses. Finally, the visual inspection of data underscored the individualized patterns and reported differences in the family functioning reports across parents and adolescents.

**Conclusion:**

Our findings emphasize the value of EMA in studying family (eg, adolescent-caregiver) behaviors. EMA’s ability to capture immediate experiences presents a nuanced picture of daily interactions and offers suggestions for practice when using the EMA methodology in populations such as the one included in this study (ie, primarily Spanish-speaking parent-adolescent dyads).

## Introduction

### Background

Family-based interventions (FBIs) are effective tools for targeting several adolescent behavioral outcomes [1]. The mechanism underlying the effectiveness of FBIs is the improvement in family functioning behaviors such as parent-adolescent communication, family cohesion, and parental monitoring [2]. For example, families exhibiting strong family functioning behaviors tend to have a decreased likelihood of adolescents participating in harmful behaviors [3]. Oftentimes, FBIs target family functioning behaviors by engaging parents or primary caregivers in individual parent sessions, where parents or primary caregivers can learn healthy parenting skills, and in dyadic parent-child sessions, where they can practice these skills [4]. The effects of the intervention on family functioning are then assessed at intermittent times (eg, baseline, that is, before intervention implementation; after the intervention; 3 months post intervention; 6 months post intervention). Few FBIs measure and evaluate family functioning behaviors in a daily (momentary) manner [5]. Rather, most FBIs utilize retrospective measurements, which limit the ability to capture data that indicate when targeted messaging tailored to participants may be needed through FBIs [5].

Ecological momentary assessment (EMA) is a collection of methods involving a high frequency of assessments (eg, receiving one or more assessments each day) that asks participants to report on individual, interpersonal, or contextual factors in real time, thereby minimizing recall bias and maximizing ecological validity [6,7]. Additionally, EMA encompasses a variety of approaches, including daily diaries [7]. EMA has been implemented with adolescents as young as 9 years by using smartphone apps and SMS text messaging [8-10]. The advantages of EMA over retrospective assessments include a higher level of temporal detail that captures changes in real time, ecological validity, and reliability [7]. Additionally, constructs assessed through EMA are often dynamic and can change quickly, and it is unfeasible to use face-to-face or in-person approaches to identify optimal opportunities to intervene [11], for example, based on changing family dynamics.

Of the existing research using EMA in the context of family functioning, most are focused on studying behavior fluctuations and understanding dynamic behavioral variations [12]. Experts in the field argue that very little is known regarding daily parent-adolescent interactions [13]. Although limited, current evidence suggests that EMA is feasible to use with early adolescents [14]; however, relatively few studies include both parent and adolescent reports when applying an EMA approach [15], with even fewer assessing family functioning behaviors [16,17]. Thus, more EMA-focused research is needed to shed light on the variability in family functioning across parents and adolescents. Capturing the dynamic changes in family functioning via EMAs could lead to adaptive intervention messaging based on daily reported family functioning, which in turn influences parent-adolescent family functioning and ultimately, adolescent behavioral outcomes.

An additional limitation of existing EMA research is the limited evidence available on EMA protocols being implemented and tested for feasibility and acceptability in Spanish-speaking (monolingual and bilingual) populations (ie, English and Spanish) [18]. One previous study using EMA methodology assessed suicidal thoughts among Spanish-speaking Hispanic/Latino adults and indicated high adherence (74.5% of EMAs completed) in their sample [19]; however, that study provided participants with smartphones to complete EMAs, possibly influencing their high adherence. Further, other studies with Spanish-speaking participants have shown relatively lower adherence (40%) [20]. Existing EMA research has made recommendations related to the implementation of EMA protocols with special populations [14], including adaptations of assessment protocols. The EMA platform technology may also need to adapt to multiple languages and EMA protocols, including protocol instructions and assessments, to have better acceptability if available in the participants’ preferred language. Therefore, more studies are needed that include Spanish-speaking parents and adolescents and that describe barriers and facilitators to protocol adherence.

### Objectives

By utilizing an approach that investigates family functioning behaviors using the EMA protocol and framework, we intend to evaluate models to develop stronger tools for FBIs. This study has 3 aims: (1) to examine the feasibility and acceptability of the developed EMA protocol, (2) to examine the within- and between-variance in daily reports of family functioning, and (3) to visually inspect changes across daily family functioning for each participant.

## Methods

### Study Participants

Adolescent inclusion criteria were (1) Hispanic/Latino/Latina/Latinx descent, (2) access to a smartphone, (3) having either English or Spanish proficiency, and (4) living most of the time with the participating parent who provided consent. Parents provided informed consent, and adolescents provided assent. All recruited participants remained in the study throughout its entirety.

### Participant Recruitment

This study uses convenience sampling via dissemination of recruitment study materials to community-based organizations that primarily serve minority populations in the Dallas/Fort Worth area. Study participants were not recruited via a clinical setting, and recruitment was primarily community-based. Interested participants completed a recruitment survey and provided their contact information. Study personnel reached out to interested individuals to set up a virtual meeting to determine study eligibility, consent, and assent, and to describe study procedures (eg, use of the EMA smartphone app, compensation structure). The eligibility criteria for the study were (1) adolescent aged between 10 and 17 years, (2) reported Hispanic/Latinx descent, (3) resided in Dallas county, (4) had a primary caregiver who was also willing to participate, (5) both adolescent and primary caregiver had access to their own smartphones, and (6) read and understood either English or Spanish. Participants were enrolled in the study on a rolling basis.

### Procedures

During the initial virtual meeting, both parent and adolescent participants downloaded the LifeData smartphone app (an existing app platform that was used to deliver the daily EMAs, available via Samsung or Apple app store for free) to their personal smartphone and viewed a training video on how to complete daily EMAs. The LifeData app delivered daily assessments via in-time notifications. The delivery of daily assessments began the day after the initial virtual meeting. Thereafter, once per day, for a week (7 days, that is, 5 weekdays and 2 weekend days), participants were asked to complete a daily assessment of family function behaviors related to that day’s (ie, the day the assessment was delivered) parent-adolescent behaviors. Only one wave of data was collected. The daily assessment was relatively brief (ie, 11 questions) to reduce participant burden, and all items were available in either English or Spanish based on the participant’s preference. Both parent and adolescent participants were advised to complete the daily assessment independently. We planned to deliver one prompt per day. If no response was received after the initial prompt notification, a second automated notification was sent 15 minutes after the first to elicit a response, with 2 additional notifications in 15-minute increments sent if no response was received. If no response was received after the notifications, the assessment for that day was considered incomplete. Participant data were reviewed by study personnel daily, and if a participant appeared to have incomplete data, study personnel followed up with the participant to determine any barrier to completion. After the 7-day EMAs, participants were sent a post-EMA, consisting of open-ended questions on their experience completing the daily assessments. Participants were compensated using online gift cards. For the initial virtual visit, parents and adolescents (ie, participants) received a US $15 gift card each. Further, participants received US $3 for each daily assessment completed. Finally, participants received US $15 for completing the post-EMA. Study data were collected and managed using the Research Electronic Data Capture (REDCap) tools hosted at UTHealth Houston [21,22].

### Measures

#### Demographics

Parents reported the sex of the child, caregiver’s relationship to the child, age of the child, ethnicity, Hispanic origin, race, nationality, years lived in the United States, caregiver’s age, marital status, education, working status, and household composition.

#### Acceptability of the EMA Protocol

Both parents and adolescents reported on the acceptability of the EMA protocol by responding to several questions (both multiple-response option and open-ended questions) related to the ease of use of the smartphone app, technical difficulties experienced, understandability of questions, and so forth.

#### Feasibility of the EMA Protocol

The feasibility of the EMA protocol was assessed by examining the average amount of time taken to complete the daily assessments, the number of daily assessments completed on average across participants, and the number of reminders delivered.

#### Daily Family Functioning

For the daily assessments, items were adapted from existing family functioning measures (most were adapted from measures used in a large national cohort study [23]) to be brief and represent various dimensions of family functioning, including communication, warmth, and monitoring. The original items and adapted items are shown in Multimedia Appendix 1. Participants were to respond based on the respective day the daily assessment was delivered. Items were worded differently toward parent and adolescent participants. For example, parents received items such as “I knew where my child was today,” while adolescents received items such as “My parent(s) knew where I was today.” There was a total of 11 items asked during the daily assessments; however, for this paper, only 9 items with the same Likert scale response option were used to create an aggregate score. Response options for the 9 included items ranged from 1=strongly disagree to 5=strongly agree. Items were averaged, with higher scores indicating better perceived family functioning (Cronbach α=0.824).

### Analytical Plan

To address aim 1, we used descriptive statistics (eg, mean, standard deviation) to assess the feasibility and acceptability data. To address aim 2, we used the *xtsum* function in R Studio (Posit Software, PBC) to examine the overall, between-, and within-variance for parent and adolescent reports of daily family functioning. Additionally, we calculated the coefficient of variation (CV) for both adolescent and parent daily family functioning to enable comparison to previous reports. Finally, to address aim 3, we examined individual and difference score plots of parent and adolescent daily family functioning by using the *ggplot* function in R Studio.

### Ethical Considerations

All study procedures were approved by the UTHealth Houston Committee for the Protection of Human Subjects (HSC-SPH-23-0129). Parents were asked to consent to study procedures, and adolescents were asked to assent. Consent procedures included a virtual visit with both parents and adolescents, where a link to the consent and assent forms was shared. Parents and adolescents were then asked to read the consent or assent form (in their preferred language, either English or Spanish) and to ask questions, if any, which the study personnel could answer. All study data were deidentified prior to analyses.

## Results

The participants in this study were 8 adolescents (7/8, 88% females; mean age 11.86, SD 1.07 years) and 7 mothers (mean age 37.29, SD 3.82 years). Additional participant information is found in Table 1.

**Table 1.** Demographics of the parents (n=7) and adolescents (n=8) in this study.

	Value
Sex of child, n (%)	
Male	1 (12)
Female	7 (88)
Age of child (years), mean (SD)	11.86 (1.07)
Parent’s relationship to child, n (%)	
Mother	7 (100)
People in the household, mean (SD)	4.71 (2.21)
Parent’s age (years), mean (SD)	37.29 (3.82)
Parent Hispanic status, n (%)	7 (100)
Parent Hispanic origin, n (%)	
Mexican	5 (71)
Other Latin American or Hispanic	2 (29)
Race, n (%)	
White	3 (43)
Other race	1 (14)
Refuse to answer	3 (43)
Parent born in the United States, n (%)	
No	7 (100)
Yes	0 (0)
Parent years in the United States, mean (SD)	11.0 (6.63)
Marital status, n (%)	
Married	6 (86)
Living with a partner	1 (14)
Parent education, n (%)	
Less than high school diploma	2 (29)
High school diploma or equivalent	2 (29)
Some college or some type of degree	2 (29)
Don’t know	1 (14)

### Acceptability

Most participants rated the overall experience with the EMA protocol as either positive (4/15, 27%) or very positive (10/15, 67%). Further, all participants rated the use of the LifeData app as either easy or very easy. Most participants felt positive (5/15, 33%) or very positive (9/15, 60%) about the questions asked as part of the daily assessments and the understandability of the questions. When asked about the burden of completing the daily assessments, most rated the burden as somewhat high or high (8/15, 53%). Some participants reported experiencing problems when receiving notifications (3/15, 20%). Finally, most participants reported that they felt that they received the right number of notifications (12/15, 80%) and at the right time (13/15, 87%). When asked to provide any additional comments in the form of open-ended questions, some participants stated that the questions became repetitive across the 7-day assessment period, the daily assessments reminded parents to communicate with their children, and the overall experience of the daily assessments was positive.

### Feasibility

On average, participants spent 3 minutes and 16 seconds (SD 11 minutes and 5 seconds) completing the daily assessment once they started the survey. The day-level response rate across the 97 daily assessment notifications sent (including all the participants) was 90% (n=87). Finally, an average of 1.89 (SD 1.79) reminders were sent to remind participants to complete the daily assessment. A large portion of the participants completed the daily assessment without needing a reminder notification sent (40/97, 41%), with adolescents more likely to complete the daily assessment without a reminder (29/53, 55% of adolescents vs 11/44, 25% of parents). When asked to provide additional comments in the form of open-ended questions regarding barriers to completing the daily assessments, some participants indicated that they experienced problems with their internet connection, not receiving daily assessment notifications, having to download the smartphone app several times, and having to check the smartphone app to see if there were any incomplete daily assessments due to the lack of notifications received. Most barriers to completion were related to technical difficulties with the smartphone app.

### Daily Family Functioning Variance

**Figure 1.**
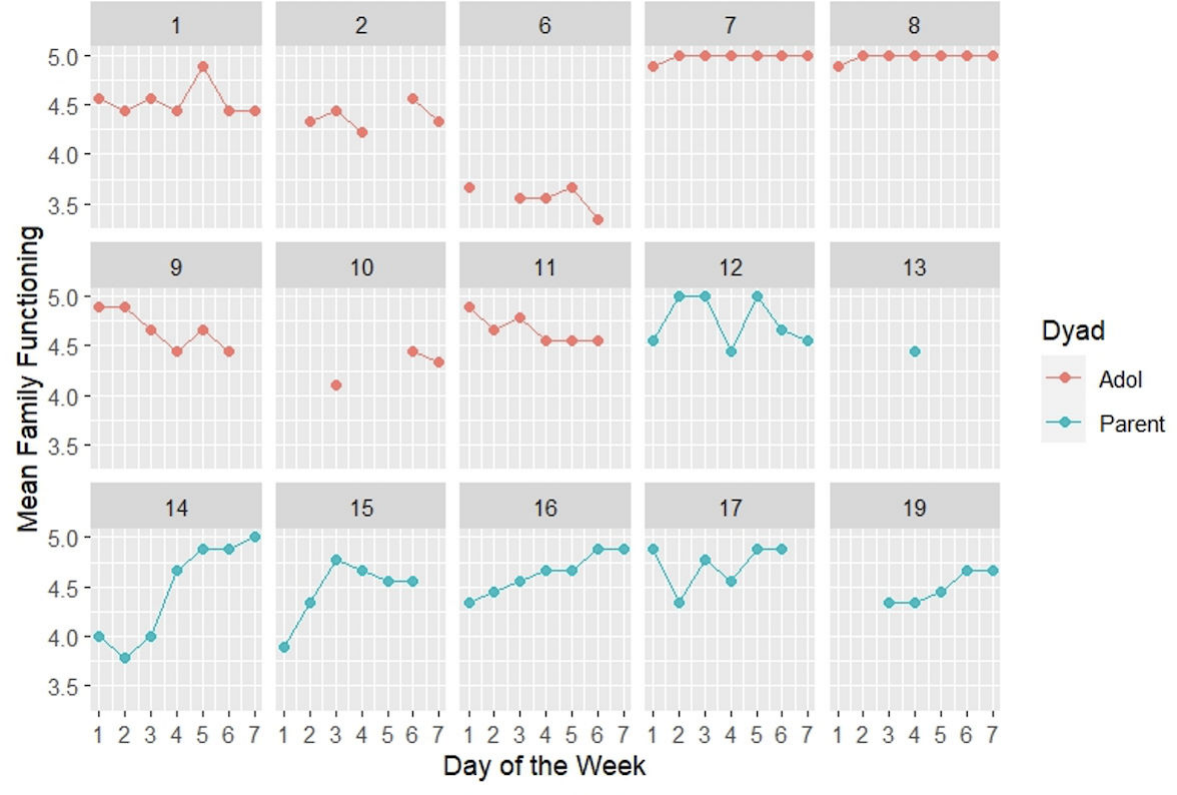
Variance in the daily family functioning across parents (n=7) and adolescents (n=8). Missing data points are daily assessments without response. Each graph represents a participant ID. Adol: adolescent.

**Figure 2.**
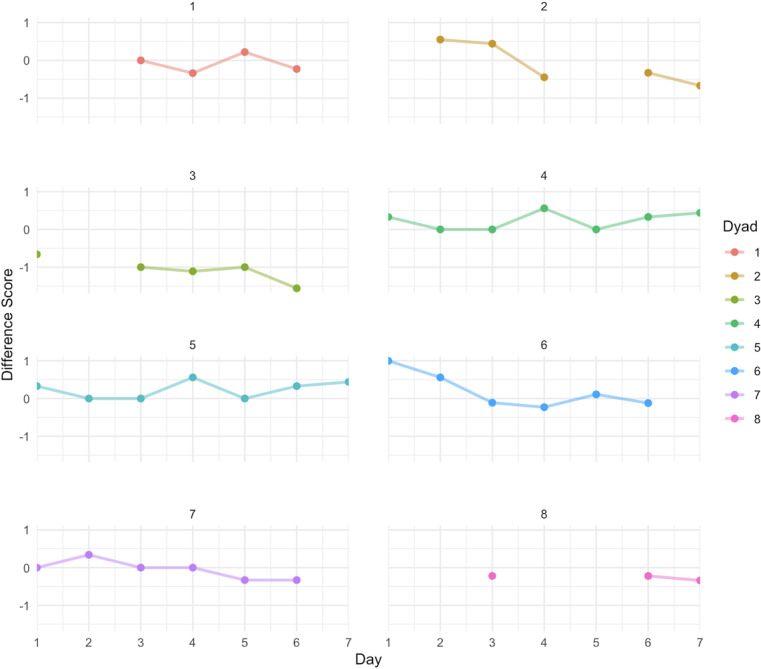
Dyadic difference scores (adolescent-parent) across 7 days. Missing points indicate that the adolescent or the parent or both had missing data for the day. Negative values indicate adolescent-reported lower levels of family functioning compared to their parents.

The reported standard deviations in Table 2 indicate that the variations in daily family functioning across adolescents (SD 0.459) are similar to the mean and standard deviations (mean 4.568, SD 0.441) observed across all person-days that had a survey response. However, when examining within-adolescent differences, adolescents were relatively more consistent in their reports of daily family functioning (SD 0.122) than when compared to the reports of the entire adolescent sample. Alternatively, across or between parents, the reported standard deviation indicated that daily family functioning was relatively more consistent (SD 0.132) compared to daily family functioning within individual parent reports of family functioning (SD 0.286), which was similar to the overall mean and standard deviation across all parents (mean 4.587, SD 0.311). The results for CV indicate a relatively low coefficient (ie, <0.10), indicating low variability and more consistent reports of family functioning for adolescents (CV=0.097) and parents (CV=0.068), with parent reports being more closely clustered around the mean when compared to adolescents. Fluctuations in the daily reports of family functioning across parents and adolescents are shown in Figure 1. Additionally, Figure 2 plots the difference scores between adolescent and parent dyads on their daily reported family functioning. Negative scores indicate that adolescents reported lower levels of family functioning compared to their parents.

**Table 2.** Overall, within-, and between-variance and coefficient of variation for parent and adolescent daily family functioning.

Adolescent or parent, variance		Value	Coefficient of variation
Adolescent daily family functioning			0.097
Overall, mean (SD)		4.568 (0.411)	
Between, SD		0.459	
Within, SD		0.122	
Parent daily family functioning			0.068
Overall, mean (SD)		4.587 (0.311)	
Between, SD		0.132	
Within, SD		0.286	

## Discussion

### Principal Findings

Our study is one of the few studies that has implemented an EMA protocol in a bilingual (ie, primarily Spanish speaking) population and has examined family functioning across parent and adolescent dyads. The purpose of our study was to assess the feasibility and acceptability of the EMA protocol in parent and adolescent dyads, examine the within- and between-variance in daily reports of family functioning, and assess changes across daily family functioning for each participant. Our findings show preliminary acceptability and feasibility among adolescents and parents in the implementation of the EMA protocol to assess family functioning; however, technical barriers related to the smartphone app hindered data collection. Additionally, findings indicated minimal variance in the reports of family functioning within participant reports.

Most participants showed a high level of acceptability toward the use of the LifeData app to administer the EMA family functioning protocol. However, it is important to note that the protocol was delivered once a day at the same time each day for a week (ie, 7 PM CST). This may have increased the burden among the participants, particularly if the set time was inconvenient for the participants. Previous EMA research with parents and adolescents implemented random notification triggers throughout a particular day [16], which may have lessened the monotony of the questionnaires and increased the chances of response across multiple time points. While considering the reported burden, overall, participants reported acceptability of the EMA protocol. Additionally, qualitatively and in response to open-ended questions, parents reported that the EMA questions served as a reminder to communicate with their children. Although previous research suggests that repeated EMA does not significantly alter behaviors [24], it may serve as a subtle prompt for parents to engage with their children [25], even if this effect is statistically minor or short-lived.

In terms of feasibility, the time to completion of the EMA family functioning protocol across participants was less than 5 minutes, and compliance was approximately 90% (87/97). These findings are consistent with those of existing EMA studies, including those implemented among youth [14,24]. A critique of EMA is the feasibility of implementing it among special populations (eg, early adolescents), with recommendations including providing proper training on EMA procedures, considering participant capabilities, adapting survey language, and scheduling accommodations, among other recommendations [14]. We applied these recommendations in our study, which may have played a role in the high compliance, while also taking into consideration the bilingual nature of our study sample. Our entire parent sample preferred assessments in Spanish, which we prepared by translating and backtranslating our assessments and providing our training materials, including our one-on-one virtual training session and training video in Spanish. Future studies implementing EMA protocols among diverse populations should make necessary adaptations to assessments as well.

In an assessment of between-participant and within-participant variance in reported family functioning, our findings suggest that the variability in reported daily family functioning varied across parents and adolescents. Although adolescents were uniform in their individual (within) reports of family functioning, parents had relatively more variation. It is possible that due to the nature of the early adolescent age (mean 11.9 years), adolescents may report consistent family functioning, as conflict may not manifest between parents and adolescents until later in adolescence when adolescents begin to explore their autonomy [26,27]. Additionally, it is important to consider the EMA protocol length (ie, 7 days). Perhaps, if the assessment length had been extended to a longer period (eg, 30 days), there may have been more variability with a probability of external factors occurring, such as academic circumstances or both immediate and extended family conflict. In the future, we intend to assess parents and adolescents by using a measurement burst design such that an EMA protocol is implemented once a month for 7 days across multiple months to allow for a more in-depth snapshot of parent and adolescent family functioning experiences.

### Limitations

Although this study provides formative evidence for the continued testing and development of an EMA protocol assessing daily family functioning, there are some limitations to consider. First, the sample size for the analysis was small (n=15; 7 mothers and 8 adolescents). The small sample size limited the variability across and between participants that may have appeared with a larger sample size. Second, most participants were recruited via a local community organization offering parenting classes, which may have influenced how the daily reported family functioning data were skewed toward more favorable reports of family functioning (skewness: adolescent=−1.16; parent=−0.77), and reports may be higher than those reported from the general public. The parents and adolescents recruited may already present better family functioning because of their potential attendance in community-based class involvement. Finally, the EMA daily assessment items were delivered at the same time (7 PM) every day on the 7 study days, which may impact recall bias. Although this was a potential limitation, our aims were primarily to assess the acceptability of the protocol (eg, English language) and the feasibility (eg, use of own smartphone for LifeData app download and use). This was our reasoning for choosing a time that would appear to be convenient for both adolescents (ie, at home from school) and parents (ie, at home from work).

### Implications

The adoption of EMA in this study highlights its practicality for real-time observation of family functioning. The variability in daily familial interactions, although relatively small, may point toward the utility of EMA in personalizing FBIs, offering a pathway for tailored strategies that align with the fluctuating dynamics of daily life. We plan to use the lessons learned in this study to fine-tune our final EMA protocol and implement within culturally responsive interventions for diverse adolescent populations and their families. Finally, these findings advocate for further research to explore longitudinal applications of EMA, potentially enhancing adolescent health outcomes through informed, dynamic support systems.

## Supplementary material

10.2196/60073 Multimedia Appendix 1 Original and adapted measures used in the ecological momentary assessment of daily family functioning.

## Abbreviations

CV: coefficient of variation
EMA: ecological momentary assessment
FBI: family-based intervention
REDCap: research electronic data capture
