# Supplementary material for: Using Ecological Momentary Assessment to Assess Family Functioning in Spanish-Speaking Parent and Adolescent Dyads: Daily Questionnaire Study
Source: JMIR Form Res. 2025 Jun 11;9:e60073. doi: 10.2196/60073 (PMC12176312; doi:10.2196/60073)
Supplement: Multimedia Appendix 1 [file formative-v9-e60073-s001.docx]

**Table S1.** Original and adapted measures used in EMA of daily family functioning.

| **Original Item** | **Adapted EMA Item for Adolescent** |
| --- | --- |
| **Parental Monitoring**  *Karoly, H. C., Callahan, T., et al. (2016) Evaluating the Hispanic paradox in the context of adolescent risky sexual behavior: the role of parent monitoring. J Pediatr Psychol 41(4): 429-40.* | |
| How often do your parents/guardians know where you are? | My parents knew where I was today. |
| How often do your parents know who you are with when you are not at school and away from home? | My parents knew who I was with (outside of school/home). |
| If you are at home when your parents or guardians are not, how often do you know how to get in touch with them? | I knew how to get in touch with my parents (if they weren’t home). |
| How often do you talk to your parent or guardian about your plans for the coming day, such as your plans about what will happen at school or what you are going to do with friends? | I talked to my parents about the upcoming day (such as what will happen at school or what you are going to do with your friends). |
| In an average week, how many times do you and your parents/guardians, eat dinner together? | My parents and I ate dinner together. |
| **Children’s Report of Parental Behavioral Inventory**  *Schaefer, E. S. (1965). Children’s Reports of Parental Behavior: An Inventory. Child Development, 36(2), 413–424.* [*https://doi.org/10.2307/1126465*](https://doi.org/10.2307/1126465) | |
| In reference to caregiver: Makes me feel better after talking over my worries with him/her. | Made me feel better after talking about my worries with him/her. |
| Smiles at me very often. | Smiled at me often. |
| Is able to make me feel better when I upset. | Was ablet to make me feel better if I was upset. |
| Believes in showing his/her love for me. | Showed his/her love for me. |
| Is easy to talk to. | Was easy to talk to. |
| **Daily Parenting**  *Janssen, L. H. C., Verkuil, B., van Houtum, L. A. E. M., Wever, M. C. M., & Elzinga, B. M. (2021). Perceptions of Parenting in Daily Life: Adolescent-Parent Differences and Associations with Adolescent Affect. Journal of youth and adolescence, 50(12), 2427–2443.* [*https://doi.org/10.1007/s10964-021-01489-x*](https://doi.org/10.1007/s10964-021-01489-x) | |
| Throughout the day, how warm/loving was your mother/father toward you? | My parent(s) were warm and supportive today. |
